# Enlightening Nursing Care: A Protocol for a Multicenter Observational Study Measuring Nursing Work in Hospital Settings

**DOI:** 10.3390/healthcare13020167

**Published:** 2025-01-16

**Authors:** Annamaria Bagnasco, Marco Di Nitto, Ilaria Marcomini, Rosaria Alvaro, Loreto Lancia, Duilio Fiorenzo Manara, Laura Rasero, Gennaro Rocco, Valeria Caponnetto, Manuele Cesare, Yari Longobucco, Francesco Zaghini, Paolo Iovino, Alessandra Burgio, Paolo Landa, Milko Zanini, Maurizio Zega, Giancarlo Cicolini, Walter Sermeus, Jonathan Drennan, John M. Welton, Beatrice Mazzoleni, Loredana Sasso

**Affiliations:** 1 Department of Health Sciences, University of Genoa, Via A. Pastore 1, 16132 Genoa, Italy; annamaria.bagnasco@unige.it (A.B.); marco.dinitto@unige.it (M.D.N.); milko.zanini@unige.it (M.Z.); l.sasso@unige.it (L.S.); 2 Centro di Eccellenza per la Ricerca e lo Sviluppo dell’Infermieristica (CERSI-FNOPI), 00184 Rome, Italy; rosaria.alvaro@gmail.com (R.A.); loreto.lancia@univaq.it (L.L.); manara.duilio@unisr.it (D.F.M.); l.rasero@unifi.it (L.R.); genna.rocco@gmail.com (G.R.); 3 Faculty of Medicine and Surgery, Vita-Salute San Raffaele University, Via Olgettina 58, 20132 Milano, Italy; marcomini.ilaria@unisr.it; 4 Department of Biomedicine and Prevention, Faculty of Medicine, University of Rome Tor Vergata, Via Montpellier 1, 00133 Rome, Italy; francesco.zaghini@uniroma2.eu; 5 Department of Clinical Medicine, Public Health, Life and Environmental Sciences, University of L’Aquila, Piazzale Salvatore Tommasi 1, 67100 L’Aquila, Italy; valeria.caponnetto@univaq.it; 6 Department of Health Sciences, University of Florence, Viale Pieraccini, 6, 50139 Florence, Italy; yari.longobucco@unifi.it (Y.L.); paolo.iovino@unifi.it (P.I.); 7 Centre of Excellence for Nursing Scholarship, c/o OPI Roma, Viale degli Ammiragli 67 sc. B, 00146 Rome, Italy; manuelecesare@gmail.com; 8 Degree Course in Nursing, Catholic University “Our Lady of Good Counsel”, 1000 Tirana, Albania; 9 Istituto Nazionale di Statistica—ISTAT, Via Cesare Balbo, 16, 00184 Roma, Italy; burgio@istat.it; 10 Département des Opérations et Systèmes de Décision, Faculté des Sciences de L’administration, Université Laval, Quebec, QC G1V 0A6, Canada; paolo.landa@fsa.ulaval.ca; 11 Axe Santé des Populations et Pratiques Optimales en Santé, Centre de Recherche du Centre Hospitalier Universitaire de Québec, Quebec, QC G1V 2M2, Canada; 12 Groupe de Recherche en Écologie Buccale, Université Laval, Quebec, QC G1V 0A6, Canada; 13 Centre Interuniversitaire de Recherche sur les Réseaux D’entreprise, la Logistique et le Transport (CIRRELT), Quebec, QC G1V 0A6, Canada; 14 Isola Tiberina Hospital—Gemelli Isola, A. Gemelli IRCCS University Hospital Foundation, Largo A. Gemelli, 8, 00168 Rome, Italy; maurizio.zega@fbf-isola.it; 15 Federazione Nazionale Ordini Professioni Infermieristiche (FNOPI), Via Agostino Depretis, 70, 00184 Rome, Italy; g.cicolini@unich.it; 16 Department of Innovative Technologies in Medicine & Dentistry, “G. d’Annunzio” University of Chieti—Pescara, 66100 Chieti, Italy; 17 Leuven Institute for Healthcare Policy, KU Leuven Kapucijnenvoer 7 Blok g, P.O. Box 7001, 3000 Leuven, Belgium; walter.sermeus@kuleuven.be; 18 UCD School of Nursing, Midwifery and Health Systems, University College Dublin, D04 V1W8 Belfield, Ireland; jonathan.drennan@ucd.ie; 19 Division of Health Systems, Leadership, and Informatics, University of Colorado College of Nursing, Aurora, CO 80045, USA; john.welton@cuanschutz.edu; 20 Department of Biomedical Sciences, Humanitas University, Via Rita Levi Montalcini 4, 20090 Milano, Italy

**Keywords:** inpatients, nursing research, healthcare costs, economic models, diagnosis-related groups, nursing records

## Abstract

**Background**: Rising costs and demands for improved quality of care present complex challenges for existing healthcare systems. The strain on healthcare resources is exacerbated by the increasing complexity of patient conditions. The Diagnosis-Related Group (DRG) system classifies inpatients according to clinical and treatment criteria, controls healthcare expenditures, and ensures the sustainability of procedures. The cost of nursing care is included in the DRG system in the same way as other fixed costs of hospital care, but the amount of nursing care provided for the same DRG can vary widely. This study, which is based on this protocol, will aim to assess the variability of nursing costs within and across DRGs and to measure how much variability in nursing care is explained by DRGs by comparing nursing care delivery in acute care hospitals with the DRG reimbursement system in Italy. It is necessary to develop a specific protocol to ensure systematic and consistent data collection at the national level. **Methods**: A multicenter retrospective cross-sectional study will be conducted. A random sample of five public Italian hospitals will be enrolled. Patients included in medical or surgical DRGs, hospitalized and discharged in 2022 will be included. Data will be collected retrospectively from two sources: hospital discharge records and nursing records. Inferential statistics will be used to assess the variability of nursing time and costs across hospitals and DRGs. Nursing costs will be determined by several factors, including time spent on nursing activities and the hourly wages of nursing staff. The time needed to complete each activity will be estimated by a convenience sample of nurses from the hospitals included in this study. The annual salary of nurses will be used to calculate the nursing cost per minute, multipled by the amount of time spent per each nursing activity. The cost per patient per day of hospitalization will be calculated. **Conclusions**: The results of this study will shed light on the variation in nursing care across different DRGs. This understanding will guide recommendations for optimizing healthcare resource allocation and enhancing the efficiency of the DRG system in Italy.

## 1. Introduction

The current healthcare system is facing a complex challenge, with rising costs and the need for improved quality of care [1]. This is exacerbated by the growing complexity of patient conditions, particularly chronic illnesses and an aging population, which put pressure on healthcare systems [2].

To maintain the sustainability of public finances, it is crucial to monitor public health expenditure. Governments reimburse health service expenditure through specific reimbursement systems, such as the Diagnosis-Related Group (DRG) system. The DRG system was created to maintain sustainable inpatient procedures, control healthcare costs, and categorize inpatients based on clinical and treatment criteria. Patients in the same group are expected to use similar resources, encouraging clinicians to effectively manage costs [3,4]. However, this system does not include the costs of nursing care [5].

Nurses are the largest professional group in the healthcare system. However, very little is known about the cost and quality of nursing care, and more specifically, the effectiveness of the care provided by each nurse [5]. In almost all countries, nursing care costs are considered a service cost and are included in the DRG system, similarly to hospitals’ other fixed costs [3]. The DRG system estimates the cost of hospital nursing care based on the total number of nurses assigned to inpatients. Consequently, nursing care costs are calculated as an average lump sum that does not take into account care intensity, the level of patient dependency, the variability in nursing resource consumption, and the intrinsic and intangible characteristics of nursing care [6]. This implies that the amount of nursing care may vary considerably even within the same DRG [7].

Previous studies have contributed to the definition of new reimbursement models in the attempt to highlight the cost weights of nursing care [7,8,9,10]. In the payment rate formulation process theorized by Knauf [7], nursing costs were assigned to each DRG based on nursing intensity weights (NIWs). This model was developed using a Delphi approach and was capable of interpreting the quantity and types of nursing activities provided to patients. Welton and colleagues tested the ‘nursing intensity billing model’ by establishing a fixed nursing weight per diem for nursing intensity (i.e., nursing hours and costs). The results highlighted that the addition of a single nursing component that was not previously included in inpatient reimbursement systems improved hospital payment accuracy. Dykes and colleagues estimated the time spent on nursing activities through relative value units (RVUs) using nursing assessment data from the electronic health record (EHR) [8]. Time-Based Activity Driven (TBAD) costing is an alternative micro-costing method developed by Kaplan and colleagues [9], which evaluated the amount of time spent in key nursing activities across the care cycle (both clinical and administrative). Sermeus and colleagues [10] theorized a reimbursement model called ‘Nursing Minimum Dataset II’ (NMDSII), which accounts for nursing resource weights by measuring the time and frequency of the nursing activities performed. Other authors have studied other variables that contribute to assessing the weight of nursing care. Griffiths and colleagues [11], for example, evaluated the impact of staffing models on patient outcomes. Staffing models with higher baseline rosters increased costs but led to better outcomes (i.e., reduction in mortality and lengths of stay). Jenkins and Welton [12] pointed out that patient severity was associated with an increase in costs and nursing intensity, while nursing experience did not significantly increase the cost of care activities.

Although several models for adequately evaluating nursing costs have been theorized, the results of these studies are still preliminary [5] and a definitive model assessing the cost weight and effectiveness of nursing care is unavailable.

In Italy, there are currently no studies that have shown whether the inclusion of nursing data in the reimbursement systems of healthcare services can contribute to a more equitable distribution of healthcare financial resources. The study based on this protocol will aim to fill this gap by examining the costs related to nursing activities within the DRG reimbursement system. The findings could clarify the variability in nursing costs provided within the same DRG, offering insights on how to better align DRG reimbursements with actual nursing care costs. Achieving this alignment may involve adjusting the DRG cost weights to account for nursing care or establishing a separate nursing intensity weight for each DRG category. Moreover, the results could help develop a replicable framework in this and other settings for assessing nursing costs and quality of care and further examine the relationship between care, patient outcomes, and healthcare costs.

## 2. Materials and Methods

### 2.1. Aims

The primary aims of this study are (1) to assess the variability of nursing costs between different DRGs and within the same DRG; and (2) to measure how much variability in the intensity of nursing care is explained by DRGs.

The secondary objectives of this study are (1) to measure the amount of nursing activity in acute care hospitals; (2) to examine the variability of the intensity of nursing care activities by DRG within patients and across patients controlling for hospital and region; and (3) to identify and quantify any differences in care needs among patients discharged with the same DRG.

### 2.2. Study Design

A multicenter retrospective cross-sectional study will be conducted. A convenience sample of all patients admitted and discharged in the year 2022 will be collected. Data will be collected retrospectively from two sources: (1) hospital discharge records and (2) nursing records. Retrospective data collection will offer a comprehensive picture of the nursing care delivered, as well as the possible heterogeneity of information on nursing activities provided by various healthcare institutions. The discharge records will be used to extract patient demographic data (e.g., gender, residency) and clinical data (e.g., diagnosis, diagnostic procedures performed, and length of stay). Nursing care activities (i.e., frequency and type) will be collected from nursing records. These data will be extracted from the EHR systems of each participating hospital.

### 2.3. Study Setting

A random sample of 5 public acute hospitals will be selected from the total number of potentially eligible hospitals that have expressed interest in participating in this study. To ensure representativeness across Italy, one hospital will be chosen from each geographical area identified according to the classification of the Italian National Institute of Statistics (ISTAT):

1. Northwest (Liguria, Lombardia, Piemonte, and Valle d’Aosta);
2. Northeast (Emilia-Romagna, Friuli-Venezia Giulia, Trentino-Alto Adige, and Veneto);
3. Central (Lazio, Marche, Tuscany, and Umbria);
4. South (Abruzzo, Basilicata, Calabria, Campania, Molise, and Puglia);
5. Islands (Sicily and Sardegna).

To ensure randomization when selecting the sample of hospitals, a comprehensive list of all public hospitals that expressed interest in participating in this study across the five geographical areas of Italy will be created. Then, we will use stratified random sampling to randomly select one hospital from each area, employing a random number generator to eliminate bias.

### 2.4. Eligibility Criteria for Hospitals

The list of Italian hospitals published by the Ministry of Health [13] will be consulted. To be eligible for enrolment, hospitals need to meet the following inclusion criteria: (1) have more than 200 beds; (2) be a public hospital; (3) have at least two wards belonging to medical and surgical departments; (4) provide written consent to participate in the study; and (5) have had an electronic nursing documentation system in place since 1 January 2022. Only medical and surgical departments will be considered, as we expect greater variability in nursing activities per DRG in these settings. Clinical units that treat acute conditions for less than one day, such as emergency rooms and day hospital wards, as well as maternity units, will be excluded. Healthcare services listed by the Ministry of Health as nursing homes or rehabilitation facilities will also be excluded from this study.

### 2.5. Eligibility Criteria for Patients

All data regarding included patients admitted and discharged in the year 2022 will be collected. Data will be collected from patients who meet the following inclusion criteria: (a) patients with medical or surgical DRGs (selected by the department code on the hospital discharge form) with a length of stay > 1 day; (b) patients whose admission and discharge took place in the same ward; and (c) patients admitted and discharged in 2022. Patients aged < 18 years will be excluded.

### 2.6. Study Variables

The variables that will be collected are listed in Table 1.

Comorbidity will be assessed using the Cumulative Illness Rating Scale (CIRS), a validated tool used for hospitalized geriatric patients [14]. The disease severity score, ranging from 0 to 4, is based on 14 components reflecting organs impacted by a chronic disease. The CIRS Severity Index measures the number of items with disease severity ratings of three or four. This index is measured on hospital discharge forms at a national level.

To assess the complexity of patients, the score of the Corridor Triage (Tri-Co) instrument will be collected. Tri-Co is composed of the Modified Early Warning Score (MEWS) [15] and the Index of Care Dependency (IDA) [16]. The first tool collects data on clinical stability/instability, while the second one classifies information on care complexity. The integration of those two instruments is justified by the concept that clinical instability and care complexity should naturally complement each other to correctly classify patients according to their level of care intensity and ensure that the provision of care is proportional to the patient’s clinical complexity [17]. Tri-Co has been previously evaluated in several Italian settings [18,19,20]. If Tri-Co is not used in the hospital or wards included in this study, considering the expected heterogeneity in care complexity assessment among hospitals, other validated tools for measuring complexity will be considered. For example, the National Early Warning Score (NEWS), the Index of Care Intensity (IIA), or the Index of Care Complexity (ICA) may be considered [20]. All these measures will be collected as single measures at the beginning and at the end of the patient’s stay and specifically at the time of patient’s admission and the last measurement before discharge, as these assessments are made by nurses to ensure adequate nursing planning.

### 2.7. Procedures

This study started in January 2024 and is planned to end in March 2025. First, a pilot study was conducted to assess the characteristics of the EHR in the enrolled hospitals. Given the lack of a unique national electronic system for storing health information, heterogeneity in the collection and organization of the nursing activities is expected. Therefore, a preliminary meeting was organized with the nursing directors of each hospital. During this meeting, nursing directors described the EHR used in their hospitals. From this information, an electronic dataset was prepared to allow each hospital to enter information on nursing care activities. For each hospital, two medical and two surgical wards will be considered for enrolment. If there are more than two medical/surgical wards in the same department, the two wards with the highest number of patients admitted during the study period will be selected.

Eligible patients will be enrolled by searching the EHR. Data will be collected retrospectively to gather information on nursing care provided in 2022. This study will collect data from all patients included in medical or surgical DRGs. All nursing activities performed on these patients and documented in the EHR will be considered.

Within each hospital, a procedural manager will be designated to extract and de-identify data of interest. The data of patients who meet the inclusion criteria will be transmitted to the principal investigator (PI) by uploading the data into a secure server specific for this study. The upload will take place via a dedicated cloud platform established on an ad hoc basis for the project, allowing each local investigator of the participating centers to access and provide data.

**Figure 1:**
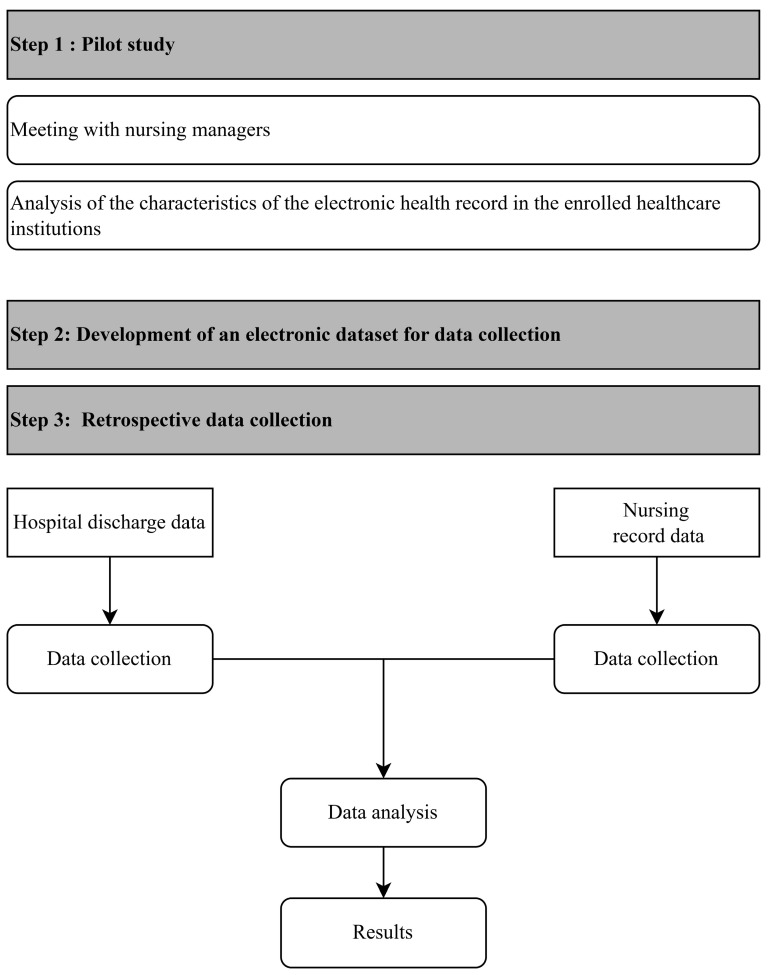
Study phases.

For login purposes, each local investigator will have a username and password (which can be modified by the user after the initial login). Each local investigator will only have access to his or her own uploaded data, while the principal investigator will have access to all the data submitted by each participating center. The Italian Regulatory Board for Nursing Professions (FNOPI) owns the server where the data will be kept. Data gathering will be continuously evaluated, and all disaster recovery measures will be in place. Figure 1 summarizes the steps of the study.

### 2.8. Data Analysis

Descriptive statistics (frequency, percentage, mean, median, range, and standard deviation) will be used to describe the sample. *t*-tests, ANOVA (or related nonparametric tests in case of a non-normal distribution of dependent variables), linear regression, and quantile regression will be performed to evaluate the possible variability in nursing time across hospitals and DRGs.

The chi-square test will be used to analyze any differences in the average frequency of nursing activities within the same DRG. In addition, we will perform linear, logistic and quantile regression, considering cost as the outcome of nursing care. Nursing costs will be assessed across hospitals and DRGs. The quantile regression will be used to explore which factors are associated with the different quantiles of nursing costs.

Nursing costs will be calculated based on several factors, including the average time required to perform each procedure, nurses’ hourly wage, the cost of materials used, and the average length of patient stay. Nursing time will be estimated using a convenience sample of nurses from the hospitals included in this study. For each nursing activity, the participating nurses will indicate the average time spent on the activity and devices relevant to each intervention. Nursing costs will be calculated by applying nurses’ salaries, based on national cost tables for nursing professionals. The annual nursing salary will be used to estimate the cost per minute of nursing time. This cost per minute will then be multiplied by the amount of time spent on each nursing activity and by the total number of activities performed for each patient. Additionally, the cost of devices required to perform each activity will be included, based on prices from the national price list. Thus, the cost per patient per day of hospitalization will be calculated. This cost will enable us to compare patients with different lengths of stay. Finally, subgroups based on patient complexity will be considered.

Moreover, nursing costs will also be compared between patients with chronic and acute diseases. This will allow us to explore the hidden nursing costs derived from the nursing care delivered on an ongoing basis for chronic diseases (e.g., symptom monitoring, administration of medication for the chronic disease, etc.) that did not show signs of exacerbation during hospitalization. A subgroup analysis will be considered to determine which patients require more nursing care (in terms of time and costs). The nursing case mix (i.e., the patient characteristics that best explain the amount and costs of nursing care) will be evaluated to provide evidence for separating out nursing care activities and nursing intensity in the Italian prospective payment system.

To enable accurate data analysis, the need to normalize scores obtained from different rating scales measuring the same outcome will be evaluated. If information from the retrieved nursing records is missing, a missing value analysis will be performed to assess the appropriateness of multiple imputation [21]. Specifically, the reason for missingness will be examined, followed by the analysis of fraction of missing information and assessment of auxiliary variables to be used for multiple imputation. Then, sensitivity analyses involving several imputation models may be performed based on missing value analysis. Patients with missing data from all variables retrieved from the nursing record (i.e., missing data on nursing activities, nursing assessment and patient complexity) will be excluded. All descriptive and inferential analyses will be performed using R V. 4.4.2 [22] or similar software. The level of significance for all analyses will be fixed at *p* < 0.05.

### 2.9. Source of Bias

The potential sources of bias in this study will include selection bias due to the proposed convenience sample, which will consist of five public hospitals in Italy. However, we expect to collect a substantial amount of data from patients across all geographical areas of Italy, with an estimated sample size of at least 15,000 individuals. Data collection bias may also arise from retrospective data collection, where incomplete records could affect the results, leading to inconsistencies in estimating the amount of nursing activities digitally registered and the related nursing costs. This bias will be controlled by performing multiple data imputation. Contextual biases related to institutional policies and healthcare culture may also influence nursing practices and patient care across different hospitals. Therefore, a thorough examination of institutional policies and healthcare cultures will be required to address contextual biases during data analysis. A potential source of bias is that the estimated nursing costs are based on a convenience sample of nurse estimates. This approach may not accurately capture the variability in nursing care across different settings. As a result, there is a risk of either overestimating or underestimating nursing costs, depending on how accurately nurses report the time spent on various activities. Thus, the development of standardized data collection guidelines, and the provision of comprehensive training for staff, will be essential to ensure consistency and accuracy in records, thereby minimizing measurement bias related to the reporting of nursing activity time. Finally, the implementation of ongoing evaluation and feedback mechanisms will further help identify and correct biases throughout the research process.

### 2.10. Ethical Aspects

The study obtained ethical approval from the Liguria Reginal Ethics Committee (Ref. number 602/2023, ID 13601). If the participating healthcare institutions need the approval of their respective local ethic committees, the required documentation will be provided.

Data will be processed in accordance with the General Data Protection Regulation (GDPR) [23]. Each institution will provide data for the purposes of the study, using an alphanumeric code that will not allow the research team to identify any participants. All data available from nursing records will be retrospectively extracted from the EHR for only those patients who meet the inclusion criteria. The local investigator of each included hospital will upload the database of extracted data into a specific cloud (with the necessary security measures), located on a server maintained and managed by The Centre of Excellence for Nursing Research and Development (CERSI-FNOPI), the promoter of this study. Only the CERSI-FNOPI research team will have access to uploaded data. The local investigators will be provided with a password that will give them access to their personal folders where the database can be uploaded. Moreover, the local investigators will have access only to their own folders, with the opportunity to modify the uploaded database if necessary. No local investigator will be able to access the data uploaded by the other local investigators. The study will be conducted in conformity with the principles of the Helsinki Declaration, the current norms of good practice, and local laws to ensure the maximum protection of the subjects involved. The data collection form will be password-protected. Data will be kept in an anonymous database. CERSI-FNOPI will store all anonymized data for five years.

## 3. Limitations

The limitations of the study may be related to the number of missing data from nursing records, as the data will be collected from hospitals with different nursing record systems. This could lead to sampling bias. However, appropriate handling of missing data (e.g., multiple imputation) will be considered to reduce this bias. Another limitation could be related to the choice of including a limited number of acute care wards of five hospitals in this study, without adjusting for differences in hospital size. This may limit the representativeness of the findings, as the results may not fully reflect the broader population of Italian Hospitals. However, the inclusion criteria were carefully assessed to guarantee both the feasibility of this study and the generalizability of the results. Moreover, it should be considered that this will be the first descriptive study that will present a picture of existing differences in nursing costs between different DRGs and within the same DRGs. Finally, the study’s cross-sectional design, which focuses on patients admitted and discharged during 2022, represents another limitation.

## 4. Dissemination

CERSI-FNOPI will distribute the results of this study. The results of this study will be presented at conferences as abstracts, posters, and oral presentations, and published in peer-reviewed scientific journals. The results will also be reported by CERSI-FNOPI to national and local government authorities, policy-makers, and stakeholders. Finally, the study results will be shared with participating facilities.

## Figures and Tables

**Table 1:** Variables that will be collected divided by data source.

Hospital discharge data	Patient sociodemographic characteristics
	Date of patient admission
	Date of patient discharge
	DRG^†^ number assigned for each admission
	MDC number related to assigned DRG
	Admission diagnosis ^$^
	Discharge diagnosis ^$^
	1st secondary diagnosis ^$^
	CIRS
Nursing record data	Number of nursing activities performed for the same DRG
	Type of nursing activities
	Patient-related care complexity *
	Nursing assessment ^§^

DRG^†^: Diagnosis-Related Group; MDC: Major Diagnostic Category; ^$^: based on the *International Classification of Diseases*, 9th Revision, Clinical Modification (ICD-9-CM); CIRS: Cumulative Illness Rating Scale; ^§^: intended as the assessment carried out on the patient through the use of rating scales according to the company organization; *: care complexity will be measured by means of a validated tool used by hospitals.

## Data Availability

No new data were created or analyzed in this study. Data sharing is not applicable to this article.
